# English Phrase Learning With Multimodal Input

**DOI:** 10.3389/fpsyg.2022.828022

**Published:** 2022-05-24

**Authors:** Yuanlin Huang, Zina Zhang, Jia Yu, Xiaobin Liu, Yuhong Huang

**Affiliations:** ^1^School of Foreign Studies, South China Normal University, Guangzhou, China; ^2^Xincheng Middle School, Shanwei, China

**Keywords:** multimodal input, three dimensions, English phrases, instructional video, cognitive load

## Abstract

Although multimodal input has the potential to lead to more sound learning outcomes, it carries the risk of causing cognitive overload, making it difficult to determine the exact effects of multimodal input on the second language (L2) phrase learning. This study tests the efficacy of multimodal input on L2 phrase learning. It adopts a mixed-method approach by utilizing both quantitative and qualitative data. The experimental design is a 2 × 3 mixed model, with a group [the experimental group (EG) and the control group (CG)] as the between-subject factor and time (pretest, midtest, and posttest) as the within-subject factor. A total of 66 participants were divided into two groups. All materials incorporated three aspects of phrase knowledge (form, meaning, and use), but the materials of the CG were unimodal in that they were offered only on paper, and of the EG were multimodal in that they included pictures, audio recordings, and video clips. After the treatment, a questionnaire and a semi-structured interview were given to the EG learners to explore their perceptions of using multimodal materials to learn L2 phrases. The results indicate that both groups had significant gains in learning phrases, but students with the multimodal input achieved significantly better results than those with the unimodal input. Moreover, the EG students had a generally positive attitude toward the use of multimodal resources. This study validates the efficacy of multimodal input on the acquisition of English phrases and shows that cognitive overload was avoided by sequencing the information.

## Introduction

Vocabulary is a central constituent of language, and it tends to occur in the form of multiword items (Schmitt, 2010), i.e., lexical phrases. Lexical phrases, or lexical chunks, are defined as conventionalized and recurring word combinations, which are stored in long-term memory as if they were single lexical words such as *a piece of*, *figure out*, and *over and over again* (Nattinger and De Carrico, 1992). A few researchers have suggested that knowing whole chunks is crucial for learners to attain a high level of language proficiency and fluency in a second or foreign language (Nation, 2001; Perera, 2001; Schmitt, 2010; Hou et al., 2018). With a sizeable stock of lexical phrases, second language (L2) learners can use idiomatic expressions instead of unconventional phrasal expressions that may ruin smooth communication (Eyckmans et al., 2016). In addition to its oral impact, a considerable L2 lexical phrase repertoire contributes to writing (AlHassan and Wood, 2015; Appel and Wood, 2016) and grammatical accuracy (Perera, 2001). However, it appears that mastery of lexical phrases is a challenge for L2 learners (Laufer and Waldman, 2011; Hou et al., 2018; Puimège and Peters, 2020). Several studies have suggested that L2 learners know fewer multiword items than single words (Bahns and Eldaw, 1993; Nguyen and Webb, 2017), and this lack of multiword knowledge causes a large proportion of learner errors, even for advanced L2 learners (Nesselhauf, 2003; Laufer and Waldman, 2011).

One of the reasons for this lack of multiword knowledge may be found in teaching materials. L2 textbooks provide only a limited quantity of lexical phrase repetition (Tsai, 2015). Moreover, L2 learners have inadequate exposure to lexical phrases in classrooms (Meunier, 2012). Thus, other sources of input are needed for L2 learners to improve their knowledge of English phrases (Tsai, 2015; Nguyen and Webb, 2017).

Researchers have been exploring explicit ways of enhancing the mastery of lexical phrases, including repetition (Peters, 2014), noticing alliteration (Lindstromberg and Boers, 2008), and web-based concordancing instruction (Chan and Liou, 2005). These studies were concerned with the effect of different learning techniques employed during exposure to materials, but they did not question the input material itself. In many studies on enhancing vocabulary learning, exposure to multimodal input has proven to be effective and may benefit lexical phrase learning as well. Although quite a few scholars have confirmed incidental learning of lexical phrases through viewing multimodal input (e.g., Peters, 2019; Puimège and Peters, 2019, 2020), no study has been conducted in explicit learning paradigms. As incidental learning of lexical phrases from exposure by L2 learners is rather slow (Szudarski, 2012), there is a need to supplement L2 phrase learning explicitly in the classroom (Boers and Lindstromberg, 2012; El-Dakhs et al., 2018). One possible concern is that multimodal input carries the risk of causing cognitive overload in the learners. Although previous studies have identified an overall positive effect of multimodal input on L2 lexical learning, there are still conflicting results on its exact effects (Zhang and Zou, 2021). Due to a higher cognitive load, redundant information on the same stimulus may result in a negative impact on learning (Sweller, 2005). Thus, a good balance of various modes is needed to help learners create a coherent mental image of a target item to successfully learn a phrase (Nation and Webb, 2011).

As Nation (2001) suggested, word knowledge is a multidimensional construct, including form, meaning, and use. By incorporating all three aspects of phrase knowledge into learning materials, including a multimodal one, it might be possible to improve L2 phrase acquisition. The instructional materials developed in this study are in line with this requirement, i.e., three-dimensional presentations of target phrases.

The aim of this study was to compare the efficacy of three-dimensional input in a unimodal and multimodal presentation on the acquisition of English phrases. The question is whether the integration of the three knowledge dimensions (form, meaning, and use) of L2 phrases with multimodal exposure benefits L2 phrase knowledge more.

### A Three-Dimensional Model for Phrase Learning

Celce-Murcia and Larsen-Freeman (1999) and Larsen-Freeman (2003) proposed a conceptual framework for teaching grammar, namely, three dimensions of grammar. They recommended adopting a three-prong approach, including three interconnected dimensions of grammar, i.e., the form themselves, their meaning, and the pragmatic conditions governing their use. To put it another way, grammar teaching should provide information on target items in three different aspects, namely, the form, meaning, and use. Similarly, Nation and Webb (2011) listed three aspects of knowledge of a word, each of which applies to multiword units. According to them, what is involved in knowing a phrase comprises form, meaning, and use. In recent years, several studies have investigated the effects of presenting information from a perspective of this three-dimensional model, i.e., through input that integrates some or all of these three aspects (form, meaning, and use) in various ways.

For example, contextualized vocabulary learning means presenting the context in which target words are used in a single sentence or passage (Golonka et al., 2015) and involves learners inferring the meaning of the target word from its use in the sentence or passage (Rodríguez and Sadowki, 2000). The context indicates how the target words are used and contributes to the transfer of knowledge that accompanies it (Sun and Dong, 2004), thus facilitating sound vocabulary learning. Some studies (Moore and Surber, 1992; Laufer, 2006) suggest that learners' immediate and long-term acquisition can be affected by the type of vocabulary presentation (e.g., encountering words in or out of context). In Sun's study (2004), three learning conditions were designed to examine the effects of two types of learning support on learners' vocabulary learning. The first learning condition is no support (NS). The second learning condition is a sentence-level translation (SLT). The third learning condition is a combination of contextualized learning (SLT) and decontextualized learning (target warming-up), SLT + TW. Findings revealed that the SLT + TW group significantly surpassed those in the NS and SLT groups in a word understanding test. The children in this group watched the cartoon that included 29 English sentences with the translation of each English sentence. Before watching the cartoon, the flashcards of the individual target words were presented to them, and they were asked to read each word.

Elgort et al. (2018) affirmed the value of additional form-focused engagement with L2 words, in addition to learning its meaning from context. Each target item was presented in contextual learning (i.e., presented in three informative sentence contexts, e.g., Beside the bed was a trap-door that permitted {egress} to the floor below.), with either form-focused elaboration or meaning-focused elaboration. The form-focused group outperformed the meaning-focused one on vocabulary acquisition. The results showed that form-focused treatment in conjunction with contextual word learning facilitated form-meaning mapping, thus significantly boosting the quality of lexical knowledge.

These studies have implications for incorporating the three dimensions in teaching vocabulary. However, the three-dimensional model has mainly been used as a theoretical foundation for vocabulary knowledge tests (e.g., Lu, 2013; Lee and Lin, 2019; Pavia et al., 2019; Sinyashina, 2020; Teng, 2020) rather than as input enhancement. As far as we know, no study has explored the efficacy of unimodal or multimodal material designed based on the three-dimensional model for enriching the L2 phrase items.

### Multimodal Input and Phrase Learning

The dual-coding theory (DCT) (Paivio, 1986) suggests that information is processed separately in two systems, namely, a verbal system specialized in processing language and a non-verbal system specialized in processing non-linguistic information. Based on Paivio's theory, Mayer (2001) proposed the cognitive theory of multimedia learning (CTML), arguing that the human brain processes information using two discrete channels, namely, auditory and visual. The former is responsible for processing auditory information, such as spoken words, music, and sound accompanying video, and the latter processes visual information, such as print text, still pictures, animation, and video (Mayer and Moreno, 1998). CTML states that the brain employs the two systems to encode and store information to produce mental constructs. It contends that when a stimulus contains different modes of representation, a coherent mental image is created, and it is expected to promote learning (Dubois and Vial, 2000). Thus, it is desirable to provide learners with a multimodal environment, allowing for parallel information processing, which may lead to stronger mental representations of information, hence facilitating learning outcomes (Mayer, 2009).

Le-Thi et al. (2020), for instance, investigated different ways of enhancing the mastery of formulaic language within a classroom context and found that visionary techniques, which helped learners' visualization according to the target formulaic sequences, led not only to quantitatively superior vocabulary learning but also to better retention of target items. Bisson et al. (2015) also affirmed the crucial role of multimodal input in the acquisition of L2 vocabulary. In this study, participants who had been presented with a picture recalled significantly more L2 words after a week's delay. In addition, the time spent looking at the pictures predicted the recognition and recall scores. The results demonstrated the impact of exposure to multimodal input, especially the important role that pictorial information can play in L2 vocabulary acquisition. Webb and Chang (2020) explored incidental learning of L2 two-word collocations by comparing three input modes, namely, reading, listening, and reading-while-listening. The results suggested that the last condition made the most contribution to learning collocations, while the two unimodal conditions contributed to similarly sized gains. Findings from Puimège and Peters (2020) also confirmed the incidental learning of formulaic sequences from multimodal inputs, a 1-h English-language documentary without subtitles. Taken together, these studies show that exposure to multimodal input contributes to L2 lexical acquisition, both for single words and multiwords.

Although the majority of studies have demonstrated the advantages of multimodal input (e.g., Chen et al., 2012; Hagiwara, 2015; Peters, 2019), some researchers have reported that multimodal input did not lead to more efficacious L2 lexicon acquisition, and even had an adverse impact on L2 vocabulary acquisition (e.g., Lwo and Lin, 2012; Liu et al., 2018; Warren et al., 2018), which was theoretically consistent with CLT (Sweller, 2005). CLT posits that the amount of information that can be processed at one time in working memory is limited. According to Mayer and Moreno (2003), if the information is presented too fast or is too content-dense, learners may not have enough time to develop coherent mental models in organizing the presented words and pictures. This presentation of the material is referred to as a situation with a high intrinsic load, leading to a detrimental effect on learning. In Taylor's (2005) study, for example, full captions were considered distracting for L2 learners because processing video content was already a high-load activity, and having to deal with additional input (captions) imposed a cognitive overload on L2 learners. Hence, to avoid cognitive overload, it is important to ensure that multimodal information provided to learners is not overwhelming.

To sum up, while the benefits of multimodal input are generally recognized, the conditions in which multimodal input facilitates or hurts the learning of a given phrase are less clear-cut. Processing tasks with too much information at the same time may result in cognitive overload. Therefore, this study provides multimodal input sequentially. The research suggests that a good balance of various learning modes is needed and should not impose a cognitive overload upon learners.

## Methodology

To examine the efficacy of multimodal input on L2 phrase acquisition, this study is guided by the following research questions:

RQ1: What is the effect of multimodal input on the acquisition of L2 English phrases?
RQ2: What are the students' attitudes toward the use of multimodal materials?
RQ3: Does the use of multimodal input offered sequentially in phrasal learning materials result in cognitive overload for EFL learners?

### Design

**Figure 1:**
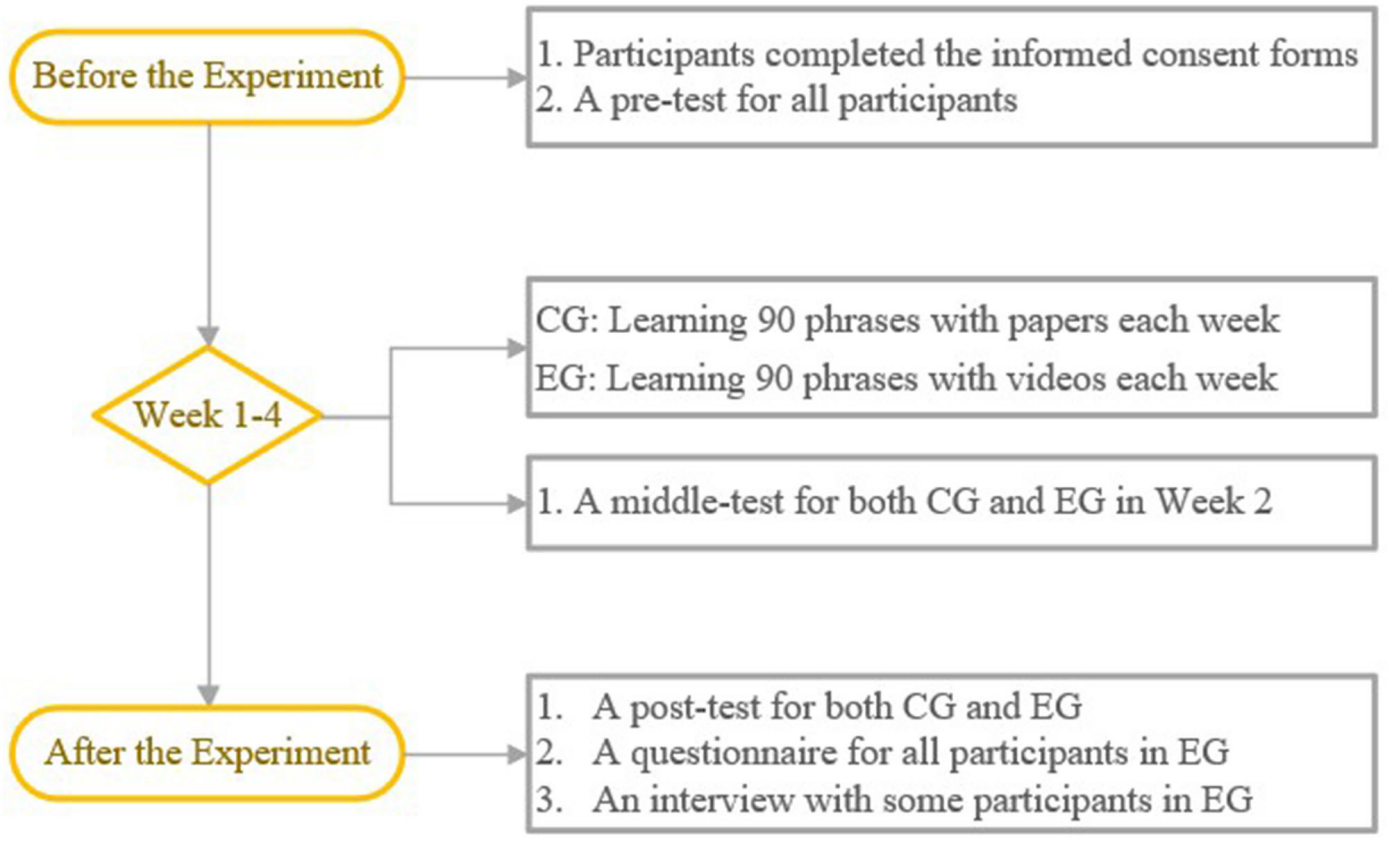
The experimental design.

Quasi-experimental methods were adopted in this study. The experimental design was a 2 × 3 mixed model, with a group [the experimental group (EG) and the control group (CG)] as the between-subject factor and time (pretest, midtest, and posttest) as the within-subject factor. The dependent variable was students' scores on phrase tests. The independent variable was the type of learning materials for EFL phrases. Two classes were randomly assigned to two groups: the CG was presented with unimodal (paper-based) EFL phrase learning materials and the EG was presented with multimodal EFL phrase learning materials. The experimental design is presented in Figure 1.

The EG group filled out a questionnaire and 10 members of the EG group were interviewed.

Notably, all tests and the questionnaire were completed through mobile phone in *wjx.cn*.

### Participants

This study occurred in two intact classes in a senior high school in Shanwei City, Guangdong Province, China. The school is located in southeast China and is one of the first-class schools in Guangdong Province. Both classes had the same teacher (one of the researchers) to teach English, and their English proficiency was approximately at the same intermediate level. The 68 participants, whose native language was Chinese, included 44 female students and 24 male students, ranging from 17 to 18 years of age. Only 66 finished all the tests, leaving each group with 33 students. Table 1 presents the information about the participants.

**Table 1:** Information about participants.

	**Male**	**Female**	**Total**
Control group	15	18	33
Experimental group	8	25	33

### Phrase Learning Materials

We selected 360 English phrases from the frequently-used phrases in the National College Entrance Examination (NCEE) in China. All sample sentences are the same in both conditions. Unimodal materials included paper-based text only (a) EFL phrases (written form), (b) the corresponding Chinese meanings (meaning), and (c) sample sentences using the phrases (use). The multimodal input contained text, audio recordings, pictures, and videos: (a) an EFL phrase (written form), (b) an audio recording of the phrase (spoken form), (c) the corresponding Chinese meaning (meaning), (d) a picture illustrating the meaning of the phrase (meaning), and (e) a video clip using the phrase (use). Audio recordings alternated between a female and a male voice and were articulated clearly and at moderate speed. The pictures were clear and illustrated the meaning of the phrases well. Video clips were chosen from a film and television corpus (www.getyarn.io) and were selected based on length, context, and meaning. How the three dimensions of form, meaning, and use of the phrases were communicated in unimodal and multimodal materials is presented in Table 2. For unimodal materials, form, meaning, and use were communicated through text mode only. Apart from text mode, the form of the phrase in multimodal materials was also given through sound mode (audio recording), and the meaning of the phrase was presented with the help of a picture. What is more, the use of the phrase was communicated through a video clip instead of text. Therefore, unimodal and multimodal materials differ only in modality and not in information.

**Table 2:** Presentation of the material based on three-dimensional theory.

	**Paper-based material**	**Video-based material**
Form	Spelling of phrase	Spelling of phrase; Audio recording
Meaning	L1 translation	L1 translation; Picture
Use	Sentence	Video (same sentence)

### Procedures

One day before the intervention classes, all participants completed a written informed consent form and finished a timed (25 min) pretest. The intervention took place in after-class sessions over 6 weeks. From Monday to Friday in the first 4 weeks, the groups were presented with either unimodal or multimodal materials.

In each session, participants in each group were required to learn the same 18 EFL phrases. Both groups spent an equal amount of time (15 min) each session on the same activities. They were asked to read aloud the phrases and the corresponding sample sentences. After reading, they also had to write down the sentences on their own in a notebook and use the phrases to make sentences of their own. These took place in the first 15 min of their English lessons, and after that, class activities began.

All participants learned 90 EFL phrases in a week and 360 phrases in total in 4 weeks. On Saturday morning in Week 2, the teacher administered a midtest to both groups. Immediately after the intervention was finished, the posttest was given.

After the posttest, the EG group was asked to fill in a questionnaire about attitudes toward using multimodal materials. A total of 10 students from the EG were randomly selected for an interview. An informal interview was carried out by the researchers through phone calls.

### Tests

To test phrase learning, three tests were compiled based on the 360 English phrases that were taught in the intervention. Each sentence contained an open slot for a target phrase with a hint in the first language. The gap-fill format assesses students' production of vocabulary (Kilikaya, 2019). Sentences for target phrases in the tests were selected from authoritative dictionaries such as the Oxford Advanced Learner's English-Chinese Dictionary and Collins COBUILD Advanced Learner's English-Chinese Dictionary. To make sure the pretest and posttest were equivalent in terms of difficulty, they elicited the same phrases, but in different sentences, which were piloted. Originally, there were 45 separate sentences in the pretest and posttest. The two tests were taken by two different groups of students who did not participate in this study and who had comparable levels of proficiency with the study participants. Because the correct rate differed greatly between the two pilot groups, 10 sentences were filtered out. Independent samples *t*-test was run to calculate the equivalence of the two tests, and the results after deletion showed non-significant difficulty differences between the two tests (*t* = −1.066, *p* = 0.292). In addition, a midtest with 30 sentences was administered to participants to check their performance during the course. The items in the midtest were developed in the same way as mentioned above. The difference between the midtest and the other two tests lies in the target phrases. The midtest contained 30 phrases selected from those phrases learned in Week 2 (90 phrases), while the pretest and posttest contained 35 phrases randomly selected from 360 phrases learned through the whole experiment.

**Figure 2:**
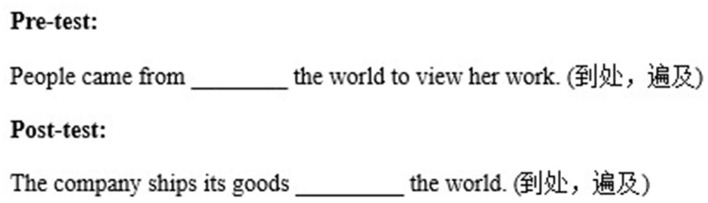
An example of pre- and post-test.

Taking the phrase “all around the world” as an example, Figure 2 shows how it was tested in the pretest and posttest. The target phrases in the midtest were selected from those learned in Week 2 and were different from the other two tests. Examples of the phrase tests can be seen in Figure 2.

### Scoring of Tests

To check participants' acquisition of the form, use, and meaning of those phrases, participants received 1 point for each correct phrase and 0 points for a wrong phrase or a misspelling. If the phrase was separated, and there were two blanks in a sentence, each blank scored 0.5 points for a correct answer. It should be noted that grammar forms were not considered incorrect as long as participants knew which phrase they should fill in and spelled it correctly. For instance, if the student response was “looking forward to” for the target response “look forward to,” it was counted as correct and received 1 point. To make scores comparable, all scores were transformed into the hundred-mark system, so the possible highest score was 100.

### Questionnaire

To examine the attitudes of participants in the EG toward using multimodal materials to learn EFL phrases, a questionnaire was adapted with reference to Sydorenko (2010). The questions involved participants' perception of phrase learning and acquisition (6 items), their evaluation of the effectiveness of multimodal materials (11 items), and their satisfaction with the videos (4 items). Each item was measured on a five-point Likert scale.

For the participants' perception of phrase learning and acquisition, the importance of phrase learning and accumulation was weighted by the participants. Besides, students also had to evaluate their knowledge of the form, meaning, and use of the commonly used phrases in NCEE.

Regarding participants' evaluation of the effectiveness of multimodal materials, items such as the effectiveness of audio recordings, pictures, and video clips in helping them master the form, meaning, and use of the phrases were included.

In terms of participants' satisfaction with the videos, items regarding the usefulness of videos in helping relieve learning stress, motivating interest, and improving confidence were included.

A question that required participants to list 5 phrases that impressed them and write down the corresponding sentences used in the video was added, which was to examine participants' phrase retention.

### Interview

A semi-structured interview in the L1 was designed and adopted to explore further what students think of the multimodal materials. The questions were developed by first asking students' common ways of learning phrases, and next comparing unimodal and multimodal methods of phrase learning and then evaluating the effects of multimodal material such as audio recordings, pictures, and videos. The questions were as follows: (1) What methods do you usually use to learn English phrases? (2) What do you think of learning phrases through Chinese meanings and sample sentences? (3) What do you think of learning phrases through Chinese meanings, audio recordings, pictures, and video clips? (4) Which of these two methods do you prefer? (5) Did pictures and video clips in the videos help you learn phrases? Please give an example to explain your reasons. (6) Did the integration of Chinese meanings, pictures, audio recordings, and video clips help you in your phrase learning? If yes, how? If no, why?

Question 1 is about the interviewees' phrase learning experience. Questions 2, 3, and 4 ask about interviewees' attitudes toward multimodal learning materials and unimodal ones. Question 5 is helpful to answer RQ3, and Question 6 is related to interviewees' satisfaction with the multimodal learning materials. Notably, the interview was conducted in the participants' native language to make sure that they could express themselves fluently. All interviews were recorded, transcribed, and analyzed.

### Analyses

The analysis used a two-way repeated-measures ANOVA with the group as the between-subject factor and time as the within-subjects factor, with a significance level of 0.05. The result of Mauchly's test of sphericity was not statistically significant (*p* = 0.814 > 0.05), which indicated that sphericity had not been violated.

Items in the questionnaire were measured in the form of a five-point Likert scale, so participants' responses were transformed into 1 point (strongly disagree), 2 points (disagree), 3 points (not sure), 4 points (agree), and 5 points (strongly agree), and scores were averaged. For the final question in the questionnaire, one point was awarded for phrases used in sentences used in the video (regarded as phrase retention) or for sentences made up by themselves (regarded as phrase use).

To address RQ3 and to gain an in-depth understanding of participants' experiences and perceptions regarding the multimodal videos, an interview was carried out in which 10 students from the EG were randomly selected (S1–S10). Interviewees' answers were recorded and transcribed with their permission, which were then summarized in terms of their feelings toward and preferences between monomodal and multimodal ways of learning new phrases and their comments on the addition of pictures and video clips. The two authors discussed each of the keywords and reached a consensus. Interveiwees' responses were analyzed to support statistical data and to answer RQ3.

## Results

### Tests

Before the intervention, a pretest was administered. In Week 2, there was a midtest. At the end of the intervention, an immediate posttest was administered. Table 3 reports the descriptive statistics from the ANOVAs with repeated measurements for both groups' performance on all three tests.

**Table 3:** Descriptive statistics of both groups' performance on three tests.

	**Pretest**	**Midtest**	**Posttest**
	**M (SD)**	**M (SD)**	**M (SD)**
The control group (*N* = 33)	15.52 (14.57)	68.79 (18.57)	48.53 (20.14)
The experimental group (*N* = 33)	16.40 (9.72)	82.93 (7.90)	70.48 (13.34)

**Figure 3:**
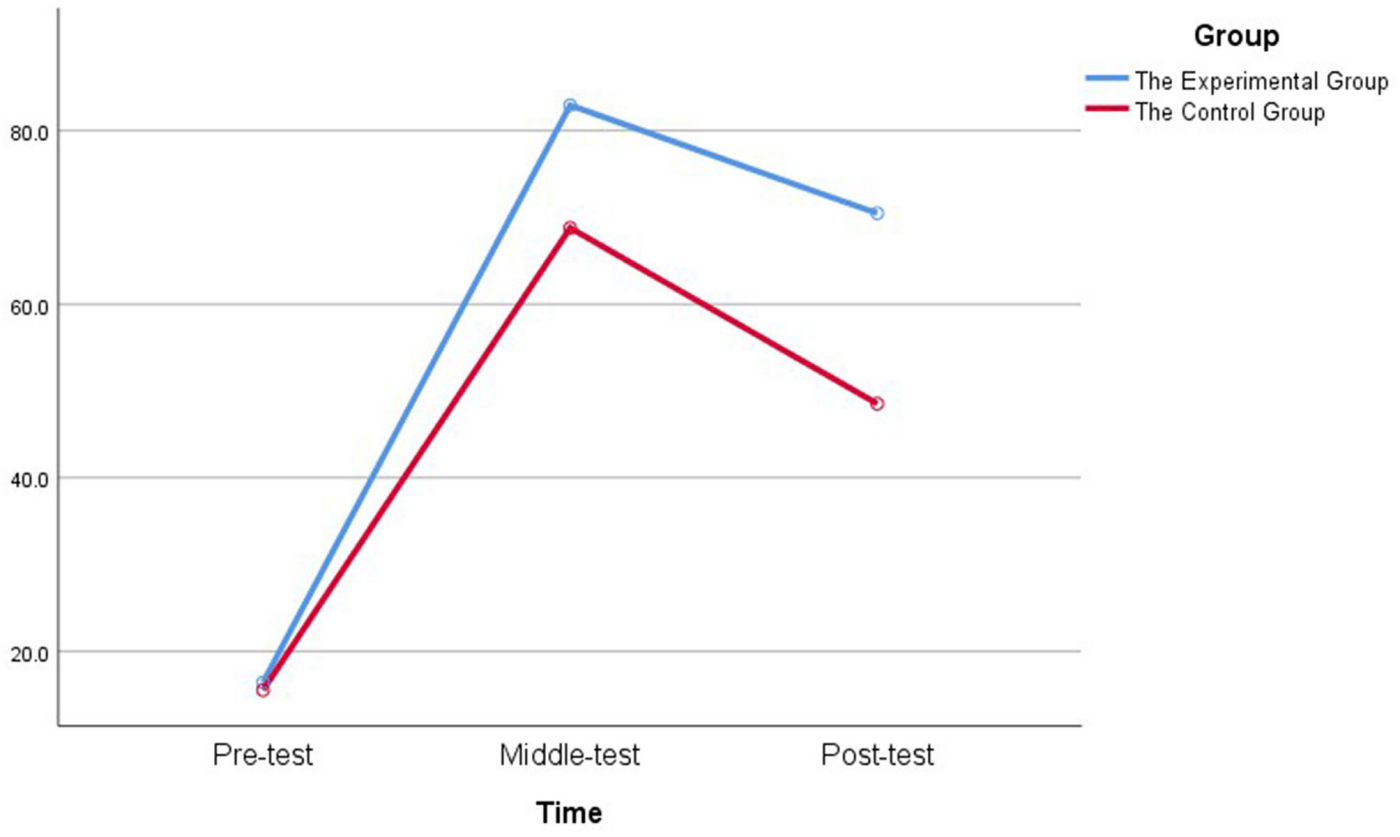
Mean scores for both groups on three tests.

Both groups made considerable learning progress on the midtest and the posttest, but the EG achieved higher scores than the CG did in both the midtest and posttest (refer also to Figure 3).

Tables 4, 5 illustrate the results of tests of within-subjects and between-subjects effects. The results revealed a significant large main effect for time [*F* (2,128) = 514.99, *p* < 0.001, $\eta_{p}^{\;2}$ = 0.89], which means the changes in participants' vocabulary pretest, midtest, and posttest results were significant. In addition, a significant large main effect for group was found [*F* (1.64) = 18.64, *p* < 0.001, $\eta_{p}^{\;2}$ = 0.23], indicating a significant difference between the two groups' performance. Besides, time and group interaction were significant on the participants' vocabulary tests [*F* (2,128) = 15.24, *p* < 0.001, $\eta_{p}^{\;2}$ = 0.19], which means that changes in participants' vocabulary tests were different between the groups.

**Table 4:** Results of tests of within-subject effects.

**Source**	**Type III sum of squares**	***df***	**Mean square**	***F***	**Sig.**	$\eta_{p}^{2}$
Time	126,530.29	2	63,265.15	514.99	0.000^***^	0.89
Time ^*^ group	3,745.42	2	1,872.71	15.24	0.000^***^	0.19
Error	15,724.49	128	122.85			

*** *p < 0.001*.

**Table 5:** Results of tests of between-subject effects.

**Source**	**Type III sum of squares**	***df***	**Mean square**	***F***	**Sig.**	$\eta_{p}^{2}$
Group	7,515.22	1	7,515.22	18.64	0.000^***^	0.23
Error	25,801.56	64	403.15			

*** *p < 0.001*.

The results of pairwise comparisons for three tests in both groups are presented in Table 6. It indicates that the scores of midtest were significantly higher than that of pretest as well as posttest in both the EG (MD mid-pre = 66.53, *p* < 0.001; MD mid-post = 12.45, *p* < 0.001) and the CG (MD mid-pre = 53.27, *p* < 0.001; MD mid-post = 20.26, *p* < 0.001), and the scores of posttest were significantly higher than that of pretest in the EG (MD post-pre = 54.08, *p* < 0.001) as well as the CG (MD post-pre = 33.01, *p* < 0.001).

**Table 6:** Results of pairwise comparisons for three tests in both groups.

**Group**	**(I) Time**	**(J) Time**	**MD (I-J)**	**Sig.**
The control group	2	1	53.27	0.000^***^
		3	20.26	0.000^***^
	3	1	33.01	0.000^***^
The experimental group	2	1	66.53	0.000^***^
		3	12.45	0.000^***^
	3	1	54.08	0.000^***^

*Number 1 stands for pretest, 2 for midtest, and 3 for posttest*.

*** *p < 0.001*.

Table 7 displays the results of pairwise comparisons for two groups in three tests. As it shows, no significant difference was found between the EG and the CG in pretest scores (*MD*_*EG*−*CG*_ = 0.841, *p* = 0.775 < 0.05). However, a significant difference was found between the two groups in midtest (*MD*_*EG*−*CG*_ = 14.14, *p* < 0.001) and posttest scores (*MD*_*EG*−*CG*_ = 21.95, *p* < 0.001), with the EG scoring higher than the CG in midtest and much higher in the posttest.

**Table 7:** Results of pairwise comparisons for two groups in three tests.

**Time**	**MD (EG-CG)**	**Sig.**
Pretest	0.875	0.775
Midtest	14.14	0.000^***^
Posttest	21.95	0.000^***^

*** *p < 0.001*.

To sum up, the results indicate that though both groups had significant gains in learning phrases, students using the multimodal video achieved significantly better results than those using the unimodal paper in the midtest and posttest. Notably, although both groups had significant losses in posttest compared with midtest scores, they still had significant gains in posttest compared with pretest scores.

### Questionnaire

The Cronbach's alpha reliability was 0.942, indicating the relevant high reliability of the questionnaire. The results are presented in Table 8.

**Table 8:** Results of the questionnaire.

**Dimensions**	**M**	**SD**
Perception on phrase learning and acquisition	4.02	0.815
Evaluation of the effectiveness of the multimodal input	4.50	0.675
Satisfaction of the multimodal videos	4.51	0.648

#### Participants' Perception and Acquisition

As Table 8 shows, participants generally appreciated the importance of phrase learning and most of them reported a good command of the form, meaning, and use of phrases (*M* = 4.02, *SD* = 0.815).

#### Participants' Evaluation of the Effectiveness

The results in Table 8 indicate an overall positive perception of multimodal resources mentioned above (*M* = 4.50, *SD* = 0.675).

#### Participants' Satisfaction

As shown in Table 8, most participants strongly agreed that the videos had helped them a lot in their learning process (*M* = 4.51, *SD* = 0.648).

#### Participants' Phrase Retention/Use

At the end of the questionnaire, participants were asked to write down five phrases that had impressed them and their corresponding sentences presented in the video. The task was completed by 17 out of 33 participants, while 15 out of 33 wrote sentences made by themselves, and one participant did not write a word, and the data were excluded from the analysis. The results are presented in Table 9.

**Table 9:** Results of phrase retention/use.

	**N**	**M**	**SD**
Phrase retention	17	3.88	1.495
Phrase use	15	4.00	1.069

As shown in Table 9, the mean scores of these items were around 4 points, which indicates that participants in the EG can either recall most of the sentences or create correct sentences by themselves.

### Interview

The interview addressed five points. Each will be discussed separately. First, with regard to the commonly used methods adopted by students to learn new phrases before the intervention, most students reported that they just look for the Chinese meaning. As S3 replied, “*I used to check the Chinese meaning when I come across a new phrase*.” But some students learn phrases differently. For instance, S1 reported that “*I used to learn a new phrase by looking it up on the Internet for its Chinese meaning and relevant pictures to get a deeper impression*.” S7 reported that “I will first look for its Chinese meaning and listen to its pronunciation, and then make up a few sentences by myself.” These suggested the potential benefits of multimodal videos in phrase learning.

Second, as to students' opinions toward multimodal phrase learning materials, all students held a positive view. For example, S4 commented that “I think this method is good and effective. I can learn a lot of phrases within a small amount of time because these resources impressed me a lot.” S8 believed that “Learning phrases with this method was interesting, especially when the clips are of great fun, which will impress me a lot.”

Third, for students' preference between unimodal material and multimodal material, 9 out of 10 showed a preference for multimodal material. For instance, S9 reported that “*The audio recordings and clips help me better memorize the phrase, and this method helps me to recall the phrase easier than the other method* (unimodal input).” In addition, others replied that “*It is more interesting than the first* (unimodal) *method and it can leave a deep impression about the phrase in the mind*.” But S2 held a neutral view of these two methods, replying that “*It depends. If I have enough time to learn, I prefer the second* (multimodal) *method. But if I don't have enough time, for example, when I am doing a reading comprehension exercise, I think the first method is also good for me*.”

Fourth, as to students' views on the addition of pictures and clips, most students showed favorable attitudes. S4, for example, reported that “*The clips definitely helped me learn phrases. Some clips were chosen from movies that I had watched, which helped me remember the phrase even more*.” S1 held a positive view on pictures, saying that “*Pictures helped me because they were colorful and vivid, which made me better understand the meaning of the phrases*.” Although most interviewees had positive views, there were a few challenges for the chosen clips. S1 commented that “*Sometimes, the meaning provided by the clips did not match with the Chinese meaning, which made me confused*.” In addition, S2 thought that “*Most of the videos were good and helpful, but I think some of them were out of fashion*.” These indicated that although the addition of pictures and clips was beneficial, there is still room for improvement.

Finally, with respect to students' evaluation of the effectiveness of multimodal material, all students thought that it was effective for their phrase learning. S5, for example, reported that “*I did not expect to remember English phrases for a long time, but the videos helped me to recall the phrases when I was doing reading or listening tests*.” S10 also commented that “*This kind of material makes the learning process become fun. It not only helped me to understand the meaning of the phrase, but also helped me in learning the pronunciation*.”

## Discussion and Conclusion

This study aimed to compare the efficacy of a unimodal and a multimodal L2 phrase learning method on EFL learners in middle school. Both approaches used a three-dimensional model based on Nation and Webb (2011), i.e., through input that integrated form, meaning, and use. In the unimodal condition, the written form of the phrases is presented in the text with words, the meaning with the L1 translation, and example sentences. In the multimodal condition, the same phrases were presented in the written form, meaning with L1 translation and example sentences with audio recordings, pictures, and a video clip (from a cartoon, movie, or TV series) in which the expression was used.

RQ1 addressed the effect of unimodal vs. multimodal L2 phrase learning materials. Based on the pretest and immediate posttest, the results showed that both methods resulted in significant gains in phrase acquisition. However, the results indicate an advantage in gains in the multimodal method.

Both methods used the three-dimensional method, parts of which confirm earlier studies: Empirical studies have shown that L1 translation is the most effective method for vocabulary learning (e.g., Moskovsky et al., 2014; Tian and Hennebry, 2016). Clear, short, and familiar definitions of L1 translations were found to speed up the process of vocabulary acquisition (Wang, 2015). Both methods used clear and short L1 translations of the phrases, which may have contributed to the learners' acquisition of the L2 phrases.

The findings are also in line with multimodal studies (Chun and Plass, 1996; Yoshii and Flaitz, 2002; Khezrlou et al., 2017; Chen et al., 2019; Ramezanali and Faez, 2019; Alzahrani and Roberts, 2021). Previous studies on multimodal input mainly focused on enhancing vocabulary learning followed to a lesser degree by listening comprehension, reading comprehension, and grammar learning (Zhang and Zou, 2021). No studies so far have examined the effect of multimodal input on phrases, and thus this study supports the effectiveness of multimodal input in promoting L2 learning. Research reveals that pictures will trigger the activation of semantic representations of words and therefore lead to efficient memorization (Bisson et al., 2015; Gruhn et al., 2020). As reported by S1 in the interview “Pictures helped me because they were colorful and they illustrated clearly and vividly the meaning, which made me better understand the meaning of the phrases.” The video clips employed in the multimodal method provided a visual context for the phrases, which helped learners to learn the meaning and use of the phrases in a rich context. S3 reported that “Videos could help me better memorize the phrase.” When learners are presented with audio, pictures, and video-based learning material, they have both auditory and visual channels stimulated, establishing auditory and visual representations of the target knowledge, enabling cognitive connections between auditory and visual representations, which are eventually committed to long-term memory to achieve higher learning efficiency (Moreno and Mayer, 2002; Mayer et al., 2014).

Moreover, it was found that the correspondence between text and imagery contributed to the acquisition of formulaic sequences (Puimège and Peters, 2019). Both pictures and video clips were carefully selected for this study and had a high correspondence to the meaning and use of the target phrases, and this might have been one of the possible reasons for the significant greater learning gains found in this study. This is in line with previous studies that have shown that “verbal + pictorial” input was more effective than verbal input alone in conveying word information because the additional pictures drew students' extra attention to word knowledge (Bisson et al., 2015). In addition, “verbal + video” input has been proven to contribute to vocabulary-learning tasks (Peters, 2019).

As for the RQ2 regarding the students' attitudes, students generally held positive attitudes toward the use of multimodal input (L2 phrase, L1 translation, picture, and video clip) for its effectiveness in learning phrases and motivating interest. As S5 mentioned, “it (multimodal input) is more interesting, and it arouses my interest to learn the phrases.” Movies and TV series enjoy high popularity among learners, which can simultaneously arouse learners' interest (Gilmore, 2007; Nooreiny and Indira Malani, 2015) and alleviate learners' anxiety (Lu et al., 2019), which can lead to better acquisition of phrases. In this study, pictures and movies arouse students' interest in phrase acquisition and provide appropriate contexts, in which learners learn the use and meaning of the phrases more effectively, and therefore contribute to better acquisition of phrases. S10 could clearly recall the phrase “think out” because it was said by one of his favorite characters in the selected video.

RQ3 dealt with the question of whether adding pictures and movie clips in phrasal learning material results in cognitive overload for EFL learners. Cognitive load theory focuses on the effects of information processing load on the construction of long-term memory (Sweller et al., 2019). In the interview conducted a month later, many participants reported that they could still recall the meanings of target phrases clearly, which indicates that multimodal three-dimensional input contributes to learners' long-term memory construction. Therefore, adding pictures and movie clips in phrasal learning material does not result in cognitive overload for EFL learners but “help gain the meaning of the phrases as a tool” as reported by S10. Plass et al. (2003) argued that multimodal input involves a higher cognitive load than unimodal input as human working memory has a limited processing capacity and will lead to less effective learning. Researchers argue that effective and efficient instructional design should minimize an unnecessary cognitive load on learners to promote better learning outcomes (Sweller, 2011). In this study, the presentation of the materials was designed based on the three-dimensional grammar theory, and in the multimodal condition, different types of information are presented in different modes. Learners process them through different channels. Since the materials were presented in a sequence, learners processed a limited amount of information at a time that did not add to the cognitive load. The three-dimensional theory could be used as a guide to structuring the presentation of phrases to EFL learners in both unimodal and multimodal materials, as both helped the learners retain the phrases well. But for greater gains, the multimodal input is advisable. Learners process different aspects of a phrase (i.e., form, meaning, and use) through different modes (see Table 2) in a stepwise fashion. These findings are not in line with Acha (2009) who found that the addition of pictures or audio to text did not result in better word-learning outcomes. However, in this study, the information was sequenced with the information given separately, the text, a translation, and then the pictures and video clips so that the information could be processed separately.

Furthermore, research showed that the simultaneous processing of two different types of information that are not automatized can lead to inadequate processing of either or both types of information (Han and Peverly, 2007). Mayer and Moreno (2003) proposed that when one channel is overloaded with essential processing demands, the cognitive load could be reduced by segmenting (allowing time between successive bite-size segments). In this study, the multimodal learning materials were presented in a sequence: form (spelling of the phrase), meaning [L1 translation, image), and use (video)]. Learners concentrate on the form and then on the meaning of the phrase, thus avoiding the influence of a “trade-off” effect.

However, Türk and Erçetin (2012) reported that the simultaneous display of multimodal information led to better performance on reading and vocabulary tests. As simultaneous presentation contributes to building referential connections between visual and verbal input, which frees up cognitive resources and makes them available for active processing. This could also explain the advantage of the three-dimensional multimodal method in this study. Learners were first presented with a form (text) and meaning (picture) so that they already understood the meaning of the phrase, which might reduce the learners' cognitive load and lead to better acquisition of the meaning of the phrase.

As for the implications of this study, first, based on the test results of the pretest and immediate posttest, both methods resulted in significant gains in phrase acquisition. Therefore, teachers can adopt different methods according to their needs. One student mentioned that multimodal input was more time-consuming than paper-based unimodal input in certain tasks. Besides, teachers would have to spend a great deal of time and effort preparing multimodal materials. It would be better if publishers could create such resources for all teachers to use. Teachers then could use multimodal three-dimensional phrases to present untaught phrases and paper-based input to help learners review learned phrases before examinations.

The learning outcome of multimodal input could be influenced by several factors: learners' working memory capacity (Acha, 2009; Gruhn et al., 2020), cognitive burden induced by multimedia input (Zhang and Zou, 2021), and conveyed information (Zhang and Zou, 2021). When the cognitive load overburdens the learners' working memory capacity, or the material is not well-designed, multimedia input may be a cognitive burden and therefore result in unsatisfactory learning outcomes (Acha, 2009; Chen et al., 2019). Accordingly, teachers should consider students' cognitive abilities and intentionally present an ideal amount of learning material to students through carefully constructed instructional design that does not overload the students' working memory.

There are several limitations to this study. First, we did not compare the gains with different types of multimodal methods, for example, learning material with text and pictures, learning material with words and video, and that with words, pictures, and video. Future studies could also address the effects of the combination of modes in a different order. Second, learners might not give equal attention to all three modes (text, picture, and video). To investigate the extent to which each of the three modes receives attention from learners, further investigation using eye-tracking techniques and its relationship with phrase acquisition is needed. Third, a delayed posttest should be carried out to examine the long-term retention. Moreover, since the cognitive load was examined using a subjective interview, future research could adopt the subjective measurement techniques as proposed by Paas (1992) or other objective measurement techniques. Finally, the result of the midtest proved that EG performed better than CG, which indicates that the multimodal method has an advantage in helping students acquire L2 phrases over than unimodal method. However, the content of the midtest is less than that of the pretest and posttest, hence not strictly comparable with the pretest and posttest.

## Data Availability Statement

The raw data supporting the conclusions of this article will be made available by the authors, without undue reservation.

## Ethics Statement

The studies involving human participants were reviewed and approved by Ethics Committee of School of Foreign Studies, South China Normal University. Written informed consent to participate in this study was provided by the participants' legal guardian/next of kin.

## Author Contributions

XbL: conceptualization, supervision, project administration, and funding acquisition. YlH, ZnZ, JY, and XbL: methodology and writing—review and editing. YlH, ZnZ, JY, and YhH: investigation. YlH, ZnZ, and JY: data curation and writing—original draft preparation. All authors have read and agreed to the published version of the manuscript.

## Funding

This research was supported by the Center for Language Cognition and Assessment, Guangdong Province, China. It's also the result of Guangdong 13th Five-Year Plan Project of Philosophy and Social Science (GD20WZX01-02).

## Conflict of Interest

The authors declare that the research was conducted in the absence of any commercial or financial relationships that could be construed as a potential conflict of interest.

## Publisher's Note

All claims expressed in this article are solely those of the authors and do not necessarily represent those of their affiliated organizations, or those of the publisher, the editors and the reviewers. Any product that may be evaluated in this article, or claim that may be made by its manufacturer, is not guaranteed or endorsed by the publisher.
